# The most frequently used tests for assessing executive functions in aging

**DOI:** 10.1590/1980-57642015DN92000009

**Published:** 2015

**Authors:** Camila de Assis Faria, Heloisa Veiga Dias Alves, Helenice Charchat-Fichman

**Affiliations:** 1,2,3 Pontifícia Universidade Católica do Rio de Janeiro, Riode de Janeiro RJ, Brasil.

**Keywords:** review, mild cognitive impairment, dementia, older adults, executive functions, neuropsychological tests

## Abstract

There are numerous neuropsychological tests for assessing executive functions in aging, which vary according to the different domains assessed.

**Objective:**

To present a systematic review of the most frequently used instruments for assessing executive functions in older adults with different educational levels in clinical and experimental research.

**Methods:**

We searched for articles published in the last five years, using the PubMed database with the following terms: "neuropsychological tests", "executive functions", and "mild cognitive impairment". There was no language restriction.

**Results:**

25 articles fulfilled all the inclusion criteria. The seven neuropsychological tests most frequently used to evaluate executive functions in aging were:

[1] Trail Making Test (TMT) Form B;
[2] Verbal Fluency Test (VFT) - F, A and S;
[3] VFT Animals category;
[4] Clock Drawing Test (CDT);
[5] Digits Forward and Backward subtests (WAIS-R or WAIS-III);
[6] Stroop Test; and
[7] Wisconsin Card Sorting Test (WCST) and its variants. The domains of executive functions most frequently assessed were: mental flexibility, verbal fluency, planning, working memory, and inhibitory control.

**Conclusion:**

The study identified the tests and domains of executive functions most frequently used in the last five years by research groups worldwide to evaluate older adults. These results can direct future research and help build evaluation protocols for assessing executive functions, taking into account the different educational levels and socio-demographic profiles of older adults in Brazil.

## Abstract (pt)

Os testes neuropsicológicos que avaliam as funções executivas no envelhecimento são muitos e variam de acordo com os diferentes domínios.

**Objetivo:**

Apresentar uma revisão sistemática dos instrumentos mais utilizados para avaliar as funções executivas em idosos com diferentes níveis de escolaridade em pesquisas clínicas e experimentais.

**Métodos:**

Foram pesquisados os artigos publicados nos últimos cinco anos, utilizando a base de dados PubMed com os termos: "neuropsychological tests", "executive functions" e "mild cognitive impairment", sem restrição de idioma.

**Resultados:**

25 preencheram todos os critérios de inclusão. Os sete testes neuropsicológicos mais utilizados para avaliar funções executivas em idosos foram:

[1] o Teste de Trilhas (TMT) Forma B;
[2] Teste de Fluência Verbal (TFV) fonológica - F, A e S;
[3] TFV categoria Animais;
[4] Teste Desenho do Relógio (TDR);
[5] Subteste Dígitos (WAIS-R ou WAIS-III);
[6] Teste Stroop e
[7] o Teste Wisconsin de Classificação de Cartas (WCST) e suas variações. Os domínios das funções executivas mais escolhidos pelos pesquisadores para serem avaliados foram: flexibilidade mental, fluência verbal, planejamento, memória operacional e controle inibitório.

**Conclusão:**

O presente estudo identificou os testes e os domínios de funções executivas mais utilizados nos últimos cinco anos, pelos principais grupos de pesquisa, para avaliar idosos. Os resultados encontrados podem direcionar pesquisas futuras e auxiliar a criação de protocolos de avaliação das funções executivas adequados aos diferentes níveis de educação e aos diferentes perfis sociodemográficos dos idosos do Brasil.

## INTRODUCTION

EFs are complex cognitive abilities that enable the identification of goals, mental planning, behavior organization, and planning actions to achieve these goals.^2,3^ In addition, EFs impact affective-emotional, motivational, and social skills.^3,4^ EFs are especially important in older adults for performing and troubleshooting routine tasks, from the most simple to the most complex. Executive functions comprise the six domains described below. Planning refers to the identification of a sequence of actions required to achieve a goal. Efficient planning includes thinking about alternatives and choosing the most effective one.^4^ Working memory is defined as a system of temporary storage and manipulation of information. This system is activated during the process of learning, language comprehension, reasoning, and production of one's conscience.^4^ Mental flexibility refers to the ability of alternating between mental sets or tasks, and changing strategies within the same task.^5^ Inhibitory control refers to the inhibition of a prepotent response, which facilitates the choice of an adequate response and avoids errors.^4^ Verbal fluency is the ability to generate an appropriate strategy for word searching.^6^ Processing speed refers to the time required to process a specific item of information.^6^ Neural substrates of these domains are mainly located in the prefrontal cortex of the brain, and are connected to several other brain areas and the central nervous system as a whole.^3^

Executive dysfunction leads to significant limitations in the daily routines of elderly, such as impact on functionality, i.e., in the performance of activities of daily living (ADLs), which reduces autonomy and quality of life.^7,8^ Deficits in EF found in older patients include inflexibility of thought, reduced working memory processing, difficulty in solving problems, decreased behavioral initiation, perseveration errors, inadequate planning strategies, and disinhibition.^1,8^ Mild cognitive impairment (MCI) is considered an intermediate state of cognitive functioning between the changes seen in healthy aging and those typically found in dementia. MCI criteria include self- or informant-reported cognitive complaints, objective cognitive impairment, preserved Independence in functional abilities and no dementia.^9,10^ Executive dysfunction in aging may appear in MCI or may be an early sign of dementia.^1^

Executive dysfunction in aging can be measured objectively with neuropsychological tests.^11^ There are several executive functions tests available, which vary according to the domains assessed. In general, a neuropsychological test predominantly assesses one of the EF domains. The wide variability of tests, the need for effective measures for early diagnosis of dementia, and the impact of executive dysfunction on ADLs, autonomy, and quality of life of older adults justify the need to identify which instruments are most used to assess executive functions in aging. This aim of this study was to present a systematic review of the instruments most frequently used to assess executive functions in older adults with different educational levels, in clinical and experimental research.

## METHODS

A systematic review was conducted on July 30^th^, 2014 using the PubMed database and combining the search terms "neuropsychological tests", "executive functions", and "mild cognitive impairment", with no language restriction. The study included articles that fulfilled the following criteria:

[1] all participants aged over 50 years;
[2] healthy older adults, MCI or dementia;
[3] neuropsychological tests used to assess executive functions;
[4] published in the last 5 years; and
[5] cross-sectional or longitudinal studies.

The studies that fulfilled the following criteria were excluded:

[1] review studies;
[2] studies with psychiatric or neurological patients, except for dementia and MCI. To assess which executive functions tests were most commonly used, a percentage of usage of over 20% was adopted as the counting criterion, considering only the articles that met the inclusion and exclusion criteria.

## RESULTS

Seventy-four articles published between July 2009 and July 2014 were found in Pubmed. Of these, only 25 fulfilled all the inclusion criteria and 49 articles were excluded according to the exclusion criteria.

Seven tests of executive function were used in more than 20% of the selected articles:

[1] Trail Making Test (TMT) Form B;
[2] Verbal Fluency Test (VFT) - F, A and S;
[3] VFT Animals category;
[4] Clock Drawing Test (CDT);
[5] Digits Forward and Backward subtests (WAIS-R or WAIS-III);
[6] Stroop Test; and
[7] Wisconsin Card Sorting Test (WCST) and its variations. The EF domains most frequently studied by the researchers were mental flexibility, verbal fluency, planning, working memory, inhibitory control, and processing speed.

Table 1 describes the relationship between the most used neuropsychological tests and their cognitive domains.

**Table 1:** The seven executive function tests most frequently used in aging research

Names of EF tests	Predominant domain	Task description
Trail Making Test (TMT) Form B	Mental flexibility	Connect 13 numbers and 12 letters alternately and as quickly as possible.^9-10^
Verbal Fluency Test (VFT) F, A and S	Verbal fluency	The test entails saying as many words a possible beginning with F, A and S in 1 minute. Participants cannot use proper nouns or use a stem word with different endings.^11-12^
Verbal Fluency Test (VFT) Animals category	Verbal fluency	The entails saying as many animal names, as quickly as possible, in 1 minute, with no restrictions on first letter or any other characteristics.^11,13-14^
Clock Drawing Test (CDT)	Planning	Draw a clock with all the numbers and pointers marking a particular time.^13,15-19^
Digits Forward and Backward subtests (WAIS-R or WAIS-III)	Working memory	In the Digits Forward subtest, the participant must repeat the numbers dictated by the examiner in the same order. In the Digits Backward subtest, the patient must repeat the same numbers in reverse order.^13,20^
Stroop Test	Inhibitory control	The test consists of three conditions: in the first condition, the subject must say, as quickly as possible, the names of the colors that are arranged on a card. In the second condition, the subject must say the names of the colors that the words “all”, “today”, etc. are printed in. In the third condition, the participant has to name the colors that the words “yellow”, “red”, etc. are printed in.^21-22^
Wisconsin Card Sorting Test (WCST) and its variants	Mental flexibility	The test consists of cards that need to be classified according to color, shape or number categories. The categorization rules change every time 10 (out of a maximum of 128) response cards have been sorted correctly. WCST variants are versions with fewer cards.^23^

Table 2 presents all the executive function tests used in the 25 articles, with their respective frequencies and percentage of usage. Table 2 also shows the number of studies by domain evaluated.

**Table 2:** Frequency of use of executive function tests in aging research.

Executive function tests	Frequency of test use	Percentage use, considering the 25 articles (%)	EF Domain	Study references (some studies used more than one test for the same domain)	Total studies evaluating the domain
Trail Making Test (TMT) Form B	9	36	mental flexibility	1, 29-42, 47	15
Wisconsin Card Sorting Test (WCST) and its variants	6	24			
Rule Shift Cards Test (BADS)	2	8			
Trail Making Test (TMT) Oral	1	4			
Intra-Extra Dimensional Set Shifting (IED) of the CANTAB	1	4			
VFT - F, A and S	7	28	verbal fluency	21-23, 32-33, 36, 38, 40, 42-46	13
VFT Animals category	6	24			
VFT Fruits category	3	12			
VFT semantics without specifying category	2	8			
VFT “S”	1	4			
VFT Vegetables category	1	4			
VFT Supermarket category	1	4			
VFT “A” of the EXIT-25	1	4			
Clock Drawing Test (CDT)	6	24	Planning	1, 21-23, 31, 33, 40, 43, 45-49	13
Rey Complex Figure Copy	3	12			
Action Program Test (BADS)	3	12			
Key Search Test (BADS)	3	12			
Zoo Map Test (BADS)	3	12			
Tower of London	2	8			
Setting Clock Test	1	4			
Verbal Clock Test	1	4			
Block Design subtest (WAIS-R)	1	4			
Raven’s Colored Progressive Matrices	1	4			
Digits Forward and Backward subtests (WAIS-R or WAIS-III)	6	24	working memory	21-22, 29-31, 33, 35-39, 42, 49	13
Codes subtest (WAIS-R or WAIS-III)	5	20			
Arithmetic subtest (WAIS-R)	1	4			
Number-Letter Sequencing subtest (WAIS-III)	1	4			
Mind Control (WMS-R)	2	8			
Clock Reading Test	1	4			
Updating Test	1	4			
SWM Strategy (CANTAB)	1	4			
Cognitive Estimation Test	1	4			
PaSMO	1	4			
Stroop test	6	24	inhibitory control	1, 22, 29-30, 38-41, 43-45, 47, 49	13
Rule Shift Cards Test (BADS)	2	8			
Inhibitory control subtest (FAB)	2	8			
D-KEFS Interference Inhibition Test	1	4			
Inhibitory control subtest (EXIT-25)	1	4			
Hayling test	1	4			
Codes subtest (WAIS-R or WAIS-III)	5	20	processing speed	30, 33, 35-37, 49	6
Simple reaction time (SRT) test (CANTAB)	1	4			

VFT: Verbal Fluency Test; BADS: Behavioral Assessment of the Dysexecutive Syndrome.

Few studies chose to use only one EF test, considering only one domain, and most studies combined two or more EF domains, as follows:

[1] Only one EF domain: 4 studies; of these, three were interested in the psychometric properties or validation of a specific test and one study opted for a mental flexibility test as the single measure of executive function;
[2] Combining two EF domains: five studies; of these, four chose one or more verbal fluency tests plus one inhibitory control test, planning test or mental flexibility test; only one study combined tests assessing mental flexibility and inhibitory control;
[3] Combining three EF domains: nine studies;
[4] Combining four EF domains: six studies;
[5] Combining five EF domains: only one study; all domains except for inhibitory control were included in this study.

These results, together with the references of the studies, are shown in Table 3.

**Table 3:** Frequency of studies by number of domains used to evaluate executive functions in aging research.

Number of EF domains evaluated in study	Domain (number of studies)	Study references
1	planning (3) or mental flexibility (1)	^1, 34, 47-48^
2	verbal fluency (4), mental flexibility (2), planning (2) and inhibitory control (2)	^23, 32, 41, 44, 46^
3	working memory (7), mental flexibility (6), verbal fluency (4), planning (4), inhibitory control (4) and processing speed (2)	^21, 29, 31, 35, 37, 39, 42-43,45^
4	working memory (5), mental flexibility (4), inhibitory control (4), verbal fluency (4), processing speed (3) and planning (3)	^22, 30, 36, 38, 40, 49^
5	mental flexibility, verbal fluency, planning, working memory, processing speed	^33^

## DISCUSSION

The systematic review presented here identified that the tests most frequently used to assess executive functions in aging research in the last five years were:

[1] Trail Making Test Form B,
[2] Verbal Fluency Test - F, A and S,
[3] VFT Animals category,
[4] Clock Drawing Test,
[5] Digits subtests (WAIS-R or WAIS-III),
[6] Stroop Test, and
[7] Wisconsin Card Sorting Test (WCST) and its variants (Table 1).

The EF domains predominantly evaluated were:

[1] mental flexibility (15 studies),
[2] verbal fluency (13 studies),
[3] planning (13 studies),
[4] working memory (13 studies), and
[5] inhibitory control (13 studies), as shown in Table 2.

Processing speed was evaluated in 6 of the 25 studies (Table 2). The assessment of processing speed is of utmost importance in aging because it is one of the first processes to decline in the early stages of dementia.^12^ However, processing speed was assessed in only six studies. Most of the selected articles favored the use of tests involving the most complex processes of executive functions that require greater action control.

As seen in Table 3, none of the 25 studies selected evaluated all six domains and only one study combined EF tests covering five domains.^33^ This can be explained by the attempt to reduce the time spent on a long neuropsychological assessment. In other words, if many neuropsychological tests were used to evaluate EF domains, in addition to tests assessing other cognitive functions, data collection would take too long, resulting in high dropout rates and making it difficult to conduct research with a large sample.^41,46^ However, important EF data is lost when some domains are not covered in a neuropsychological assessment.

There is no established criterion in the literature with respect to the optimal number of tests needed to evaluate executive functions, but, according to the results of the present review, combining 3 to 4 EF domains in comprehensive batteries was the most frequent procedure adopted.^21,22, 29,31,35-40,42,43,45,49^ The choice of three or more combinations selected by the majority of studies in this review^21,22,29-31,33,35-40,42,43,45,49^ (Table 3) is because EFs are a multidimensional construct, i.e. comprise a series of interrelated skills and high-level cognitive processing and recruit several domains in parallel, such as mental flexibility, planning, verbal fluency, inhibitory control, processing speed, and working memory.^3,4^ Researchers seem to avoid using only one or two EF measures in order to ensure that most of the EF domains are covered. A few studies^23,32,41,44,46^ (Table 3) restricted the EF assessment to two tests, usually combining a flexibility, planning or inhibitory control test with a verbal fluency test, which is quick, easy-to-apply and sensitive for discriminating people with dementia, MCI and healthy older adults.^13^

As seen in Table 2, the TMT Form B and WCST and its variants were the most frequently selected tests for assessing mental flexibility. The TMT Form B was used in nine studies (from a total of 14 studies assessing flexibility) whereas the WCST or one of its variants were used in six studies. The TMT Form B is predominantly a mental flexibility measure.^14,50^ The individual must switch their focus of attention repeatedly between two sequences (numerical and alphabetical). Mental engagement, motor dexterity, and working memory are also recruited during the test.^14,50^ The WCST and its variants also assess mental flexibility.^51^ The subject must migrate from one classification rule to another during the test. Selective attention and impulsivity are also evaluated in this test.^51^ The fact that the TMT Form B has a simpler and faster application procedure than the WCST and its variants justifies its selection by most studies.

Other tests commonly used to evaluate executive functions in aging were the VFT - F, A and S and the VFT Animals category. These tests were mainly chosen for the evaluation of verbal fluency. Seven studies that assessed this domain chose the VFT - F, A and while six chose the VFT Animals category. The VFT - F, A and S evaluate verbal fluency and entail an active search for specific information in memory.^17^ The VFT Animals category predominantly evaluates verbal fluency.^19^ During execution of the task, there is an active search for information falling into a certain category. Language and semantic memory are also evaluated in this test.^19^

Another frequently used task was the CDT, which was chosen by six studies out of a total of 13 that evaluated planning. Planning is the main cognitive process recruited during the CDT, since the circle should be large enough to accommodate the 12 numbers of the clock, which in turn should be equally distributed and correctly positioned. In addition, the hands must mark the requested time, with the minute hand greater than the hour hand. Visual constructive skills are also evaluated in this test.^18^ CDT is simple and widely used in cognitive aging research and for cognitive impairment screening in geriatric services.^18^

A working memory measure, the Digits subtest (WAIS-R or WAIS-III) was the most frequently used, having been chosen by six out of thirteen studies. This test encompasses two tasks, forward and the backward. A detailed description of the test can be found in Table 1. The Digits forward subtest (WAIS-R or WAIS-III) evaluates information storage by verbal working memory. A string of digits is dictated by the examiner and the participant has to repeat it immediately after. The Digits backward subtest (WAIS-R or WAIS-III) measures verbal content processing in working memory.

Finally, the Stroop test was the most frequently chosen test (6 out of 11 studies) for evaluating inhibitory control. It assesses the subject's ability to inhibit an automatic behavior (reading a word) and perform a controlled behavior (saying the color the word is printed in).

Executive functions undergo changes during the normal aging process, that is, performance on EF tests is expected to deteriorate with increasing age.^14,15,17-20,25-27^ Decline in EF during the aging process can be mild or severe. Older people with a significant decline in executive functions fall within the Cognitive Impairment Mild (MCI) diagnostic category and may progress to dementia.^52^ Executive dysfunction in aging is related to a reduced ability to perform activities of daily living, which creates dependency, loss of autonomy, and reduced quality of life,^7^ justifying the importance of investigating the functioning of EF in the elderly population and the main tests discussed in the present review.

Besides the age effect, EF tests are affected by education, that is, the lower the subject's educational level, the worse the test performance.^14,15,17-20,25-28^ The Brazilian older population has a higher educational variability than older people in developed countries.^53^ The relationship between education and cognition is widely acknowledged in the literature. Foss et al.^54^ conducted a study showing that educational level is strongly correlated with subtests of executive functions and other cognitive functions (attention, visual constructive skills, language, memory, and global cognition score). The sample consisted of 502 Brazilian older adults without cognitive deficits. The results indicated that illiterate older adults performed significantly worse than participants with 1 year or more of formal education. Participants with 1 or 2 years of education had a significantly worse performance on all subtests than participants with 3 or 4 years of education which, in turn, performed worse than the group with 11 or more years of formal education. Groups with 5-10 years of education showed no differences in performance.

There is evidence in the literature that the performance of executive functions in older adults is also influenced by socioeconomic status.^52^ Charchat-Fichman et al.^52^ mapped the neuropsychological profile of a sample of 88 elderly people without dementia at a geriatric outpatient clinic of a public hospital in the city of Rio de Janeiro. Patients with traumatic brain injury, stroke, Parkinson's disease, and other neurological or psychiatric disorders previously diagnosed by the medical team, were excluded. Charchat-Fichman et al.^52^ concluded that 94.3% of the sample had significant EF impairment. The researchers hypothesized that this result was a reflection of the low socioeconomic status of the sample, noting that perhaps the level of education is not the only factor involved in EF decline among elderly. Socioeconomic variables may contribute to the low performance of elderly on EF tests.^52^ These data raises the question of whether the tests commonly used to assess EF in aging are suitable for the Brazilian population.

This study identified the tests and specific domains most frequently used in the last five years to assess EF in older adults. To our knowledge, there are no other review studies in the literature on EF testing during aging. This study raises the issue of the adequacy of the most frequently used tests for use in the Brazilian older population, considering the inherent variability in educational and socio-demographic levels, which result in heterogeneity of cognitive test outcomes. Knowing which EF tests and domains are chosen by the main research groups can direct future research and help build appropriate EF assessment protocols for different educational levels and socio-demographic profiles of older adults in Brazil.
